# Impact of Early Urine Specimen Collection on Emergency Department Time to Disposition: A Randomized Controlled Trial

**DOI:** 10.7759/cureus.10495

**Published:** 2020-09-16

**Authors:** Amit Bahl, Ameen M Jamali, Gautam Ramesh

**Affiliations:** 1 Emergency Medicine, Beaumont Hospital, Royal Oak, USA; 2 Emergency Medicine, Medical Center Health System, Odessa, USA; 3 Emergency Medicine, Michigan State University College of Human Medicine, Lansing, USA

**Keywords:** ed length of stay, triage protocols, ed operations, urinalysis testing, urinalysis

## Abstract

Background

Diagnostic testing in the ED increases the length of stay (LOS). Urinalysis testing is highlighted specifically as a source of delays. We aim to determine whether a triage-initiated urine specimen collection process decreases ED time to disposition (TTD) in ambulatory patients with abdominal pain.

Methods

This prospective, randomized controlled study was implemented at a Suburban Level One trauma ED with greater than 120,000 annual visits. A convenience sample of patients was recruited. Adult, non-ambulance patients presenting with abdominal pain were eligible. Participants were randomized into experimental and control groups. Patients in the control group provided a urine sample after physician evaluation, if ordered by the provider. Patients in the experimental group were prompted to provide a urine sample in the triage restrooms immediately after screening at the greeter desk. The UA sample was transported to the treatment area and sent to the laboratory after physician evaluation.

Results

A total of 125 control patients and 124 experimental patients were enrolled. Forty-two patients were excluded because they were unable to provide a urine sample. Patients who had a urinalysis ordered were included in statistical analysis. Final data set included 65 patients in the experimental group and 96 patients in the control group. No significant difference (p=0.5072) in disposition time between subjects in the experimental group (n=65, mean=5:17 [hours:min]) and subjects in the control group (n=96, mean=5:30) was found.

Conclusions

The triage protocol for urine specimen collection did not significantly reduce ED TTD. Further research in overcrowded EDs with long patient waiting room times may benefit from implementing a triage protocol for urine specimen collection.

## Introduction

In the past 20 years, the number of visits to EDs has increased substantially. This increased number of visits has contributed to ED overcrowding nationwide [1-5]. ED overcrowding has been shown to increase the length of stay (LOS) and delay treatment [6-12]. Previous studies have identified the need for more front-end operations that can be used to decrease ED LOS and time to disposition (TTD) [13,14]. An example of such mechanism is a study by Singer et al., in which point-of-care troponin testing for patients with chest pain reduced TTD compared to patients whose troponin was measured in a central laboratory [15]. Another study investigated the impact of a triage order set for cardiac testing and found a similar reduction in TTD [16]. A third study found that a triage nurse x-ray requesting system improves the flow of the walking wounded patients in the emergency room [17]. The previous studies are examples of utilization of specific front-end operations based on chief complaint and presentation. By implementing these operations, ED TTD decreases, ED LOS decreases, treatment delays are reduced, and patient outcomes are improved [18-24].

Because diagnostic tests increase patient’s LOS, diagnostic testing time is a potential target for decreasing ED LOS [25]. In this study, urine specimen collection was investigated as a potential target for decreasing ED TTD. Urine specimen collection is patient-dependent and is highly variable in the amount of time it takes to complete. This study aimed to demonstrate that a simple triage protocol of urine specimen collection based on preselected chief complaints would decrease patient TTD.

## Materials and methods

Study design

This prospective, randomized controlled study of patients in an academic, suburban, Level One Trauma Center was conducted over a two-month period lasting from July 2014 to August 2014. Eligible participants were non-ambulance patients who were 21 years or older with a chief complaint of abdominal pain, flank pain, or pelvic pain. This study was approved by the home Institutional Review Board. Patients meeting eligibility were consented to participate in the study. Participants were randomized into experimental and control groups using an envelope system. Envelopes were prepared and sealed by a biostatistician prior to patient recruitment. The control group proceeded through the treatment process in traditional fashion, beginning at the greeter desk and culminating in the treatment area. Patients in the control group were asked to provide a urine sample after physical examination only if ordered by the provider. The treatment process of the experimental group started at the greeter desk, where patients received a urinalysis packet. Before proceeding to the treatment area, patients were prompted to provide a urine sample in the triage restrooms, which they carried with them to the treatment area. After physical examination, urine samples were sent for lab analysis, if ordered by the provider. Providers were unaware of patient recruitment into the study and were blinded to randomization arm.

Disposition order was placed into the electronic medical record by the physician, and was used as the primary outcome measure. Age, sex, heart rate, systolic blood pressure, labs, and imaging orders were examined as descriptive variables across control and experimental groups.

Data collection and processing

The patient’s status (control or experimental group), chief complaint, medical records number (MRN), and date of ED visit were recorded in a password-protected Microsoft Excel spreadsheet. Additional information, including TTD, sex, age, heart rate, labs, imaging and disposition decision (discharged or admitted) was retrieved from the hospital’s EMR system, Epic© (Cary, NC).

Outcome measure and analysis

The primary outcome measured was TTD between treatment groups. TTD was used in order to prevent factors such as increased boarding times and prolonged discharges from skewing the data. A Wilcoxon two-sample rank-sum test was conducted on the TTD between treatment groups using RStudio version 1.0.136 for Mac OS (RStudio, Boston, MA). Pearson chi-square and fisher’s exact test were performed to analyze descriptive variables across treatment groups.

## Results

**Figure 1:**
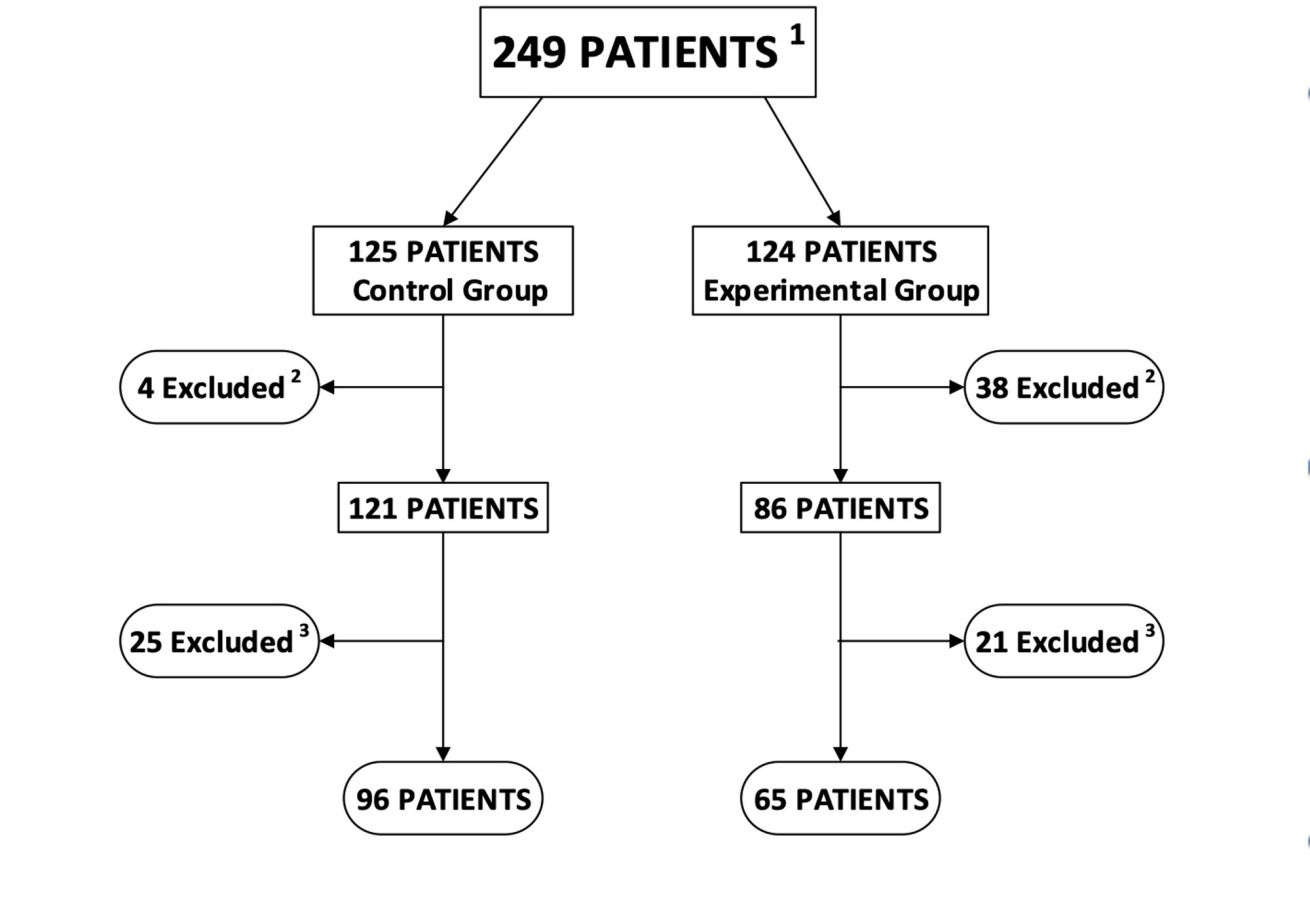
Diagram of patient flow ^1^Patients with chief complaint of abdominal pain and/or flank pain. ^2^Patients excluded as unable to provide urine sample in treatment pod bathroom in control group and triage bathroom in experimental group. ^3^Patients excluded as provider did not order urinalysis testing.

**Figure 2:**
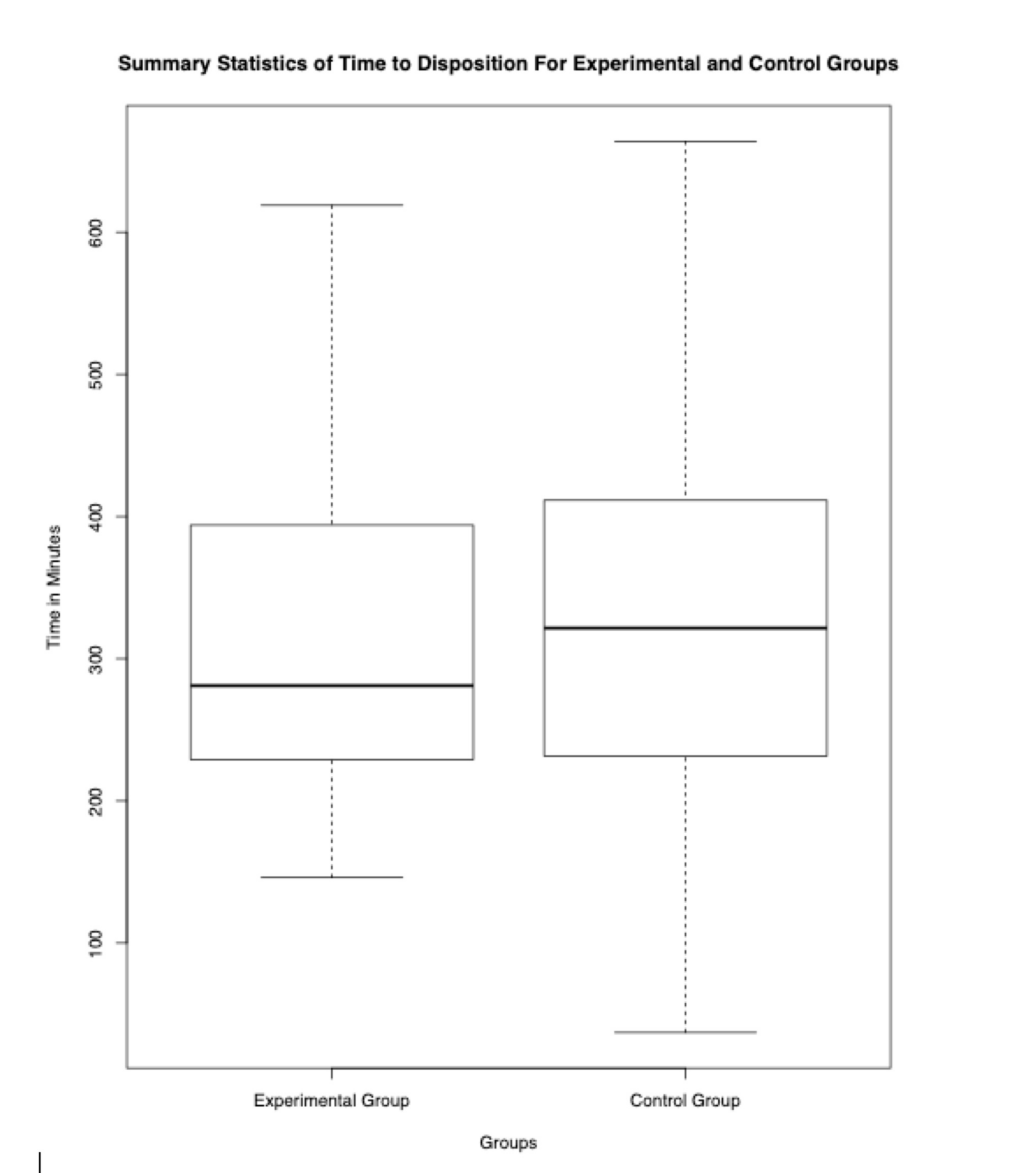
Time to disposition for experimental and control groups A Wilcoxon two-sample rank-sum with continuity correction found no statistically significant difference between the time to disposition in the experimental and control groups (p=0.5072).

Of the 249 patients recruited, 125 were randomized to the control group and 124 were randomized into the experimental group. 42 patients were excluded because they were unable to provide a urine sample leaving 121 in the control group, and 86 patients in the experimental group. Only patients who had a urinalysis ordered were included in statistical analysis. Final data set results included 65 patients in the experimental group and 96 patients in the control group, as shown in Figure 1. A Wilcoxon two-sample rank-sum test with Continuity Correction was performed on time to disposition in experimental versus control groups. No significant difference (p=0.5072) in time to disposition between subjects in the experimental group (n= 65, mean= 5:17) and subjects in the control group (n=96, mean=5:30 hours) was found. The primary outcome measure, time to disposition between experimental and control groups, is shown in Figure 2 and summarized in Table 1.

**Table 1:** Primary outcome of time to disposition for experimental and control groups

Time to Disposition								
Group	N	Mean	Std Dev	Minimum	Q1	Median	Q3	Maximum
Experiment	65	5:17	1:58	2:26	3:49	4:41	4:54	10:19
Control	96	5:30	2:19	0:37	3:51	5:21	6:51	11:04

Demographic information including age, gender, and vital signs were similar between groups. The average age was 46.41 in the control group and 42.85 in the experimental group (p=0.1911). The majority (78.12%) were female in the control group and (75.68%) in the experimental group (p=0.3515). Table 2 shows full demographic statistics.

**Table 2:** Patient demographic and clinical variables

Patient Characteristics			
	Control (n=96)	Experimental (n=65)	p-value
Mean Age	46.41 (17.7)	42.85 (16.3)	0.1911
Gender			0.3515
Male (%)	21 (21.88%)	19 (29.69%)	
Female (%)	75 (78.12%)	45 (75.68%)	
Heart Rate	86.5 (15.6)	87.7 (15.86)	0.6408
Blood Pressure	134.8 (17.0)	132.7 (15.4)	0.4273
Chief Complaint			
Abdominal Pain	85 (88.54%)	52 (80.0%)	0.1761
Flank Pain	11 (11.45%)	13 (20.00%)	
Disposition			
Admission	50 (52,08%)	29 (44.62%)	0.4417
Discharge	46 (47.92%)	36 (55.38%)	
Imaging			
Yes	87 (90.62%)	56 (86.15%)	0.5297
No	9 (9.38%)	9 (13.85%)	

Patients in both groups had similar final disposition from the ED and similar amount of imaging ordered. Approximately half of the study subjects were admitted to the inpatient wards in both groups with 52.08% and 44.62% of patients in the control and experimental arms respectively being hospitalized (p=0.4417). Imaging was similar between both groups with 90.62% and 86.15% in the control and experimental groups, respectively, receiving imaging (Table 2).

## Discussion

This study demonstrated that early collection of urine in an ambulatory ED population with abdominal pain did not improve the time to disposition. Contrary to our hypothesis, early collection of a urine sample in triage did not positively impact our ED LOS. Overall ED operational efficiency at our site likely influenced this outcome measure. Despite significant daily volumes, our ED is highly efficient with a “straight back” process so that patients spend very little time in the triage bay. Instead, patients are taken directly to the treatment bays and are quickly assessed by nursing teams and physician providers. The average door-to-doctor time was 25 minutes during our study period. The impact of the early urinalysis collection intervention may have been more relevant in a hospital with more ED overcrowding with longer patient wait times. Recreation of this study in an overcrowded ED, along with recording ED volume at the time of patient recruitment, could allow the effect of this triage urine specimen collection to be evaluated more thoroughly.

Testing in the ED has a significant impact on ED LOS. In a study by Kocher et al. analyzing 360 million ED visits, 63% of patients received some level of testing. Blood tests which included urinalysis for the purpose of this study when performed were associated with an odds ratio of 2.29 for experiencing more than a four-hour ED LOS. The most time-costly testing modalities included blood testing as the leader of the group. Optimizing delivery of emergency care requires a closer analysis of testing protocols to improve operational efficiency [25].

Front-end operational interventions such as this study have the potential to decrease ED LOS and improve the overall quality of care with downstream implications of reducing ED overcrowding and improved patient outcomes and satisfaction [18-24,26-29]. Urinalysis was chosen for this study as this data point has the potential for significant delay as it is impacted by the patient’s ability to void compared to other blood testing that is largely dependent on the practitioner taking the blood sample [25]. We felt that emphasizing the need for a urine specimen early in the ED visit would improve the time to urine collection. We were unable to find other studies evaluating early urine collection and the impact on ED LOS. Some literature exists regarding point-of-care testing with urine dipstick as a means to improve the efficiency of urinalysis testing but poor sensitivities and specificities to diagnose urinary tract infection by dipstick in several studies may limit the benefit of this type of testing [30]. Further, this intervention addresses time of testing but not the time to acquire the sample from the patient, the interval of interest in this study. Also, the point-of-care testing requires additional resources and training. 

Our study was not without some limitations. The study included only non-ambulance patients as most patients arriving via EMS do not spend time in triage at our institution. Thus the impact of a similar intervention on ambulance traffic is unclear. As these patients may have a higher severity of illness with higher admission rates, it is possible that the results of our analysis would be different. Further, we narrowed our evaluation to exclude patients with altered mental status or fever. As urinary tract infection in the elderly is a common cause of both of these complaints, early collection of urinalysis may impact ED disposition times in these subgroups. As many of these patients arrive via EMS, they were excluded from this analysis.

We recruited a convenience sample of patients on weekdays from 9 am to 5 pm. We did not analyze trends in ED volumes during recruitment or consider differences in operational efficiencies during “non-business” hours. It is unclear but our results would have been impacted by a different recruitment schedule.

Another limitation was that urinalysis was not submitted to the laboratory until the physician ordered the test rather than laboratory submission immediately after triage collection in the experimental group. While this was an extremely common test ordered in patients with abdominal pain, not all patients (approximately 20% in our investigation) required the test and we felt the decision was best left to the treating physician. More prompt laboratory submission for all patients in the experimental group may have resulted in shorter urinalysis result times and reduced ED LOS. 

A large number of patients were excluded from the analysis as a significant number of patients were unable to provide a urine sample or the provider chose not to order urinalysis testing. While the two comparative groups appeared to be similar as depicted in the analysis, the impact on the time to disposition for the randomized but excluded patients particularly on the experimental side was unknown.

## Conclusions

The triage protocol for urine specimen collection described in this study did not significantly reduce ED time to disposition. Further research in overcrowded EDs with longer patient waiting room times with patients with a more diverse range of chief complaints may provide better insight into which hospitals and populations may benefit from implementing a triage protocol for urine specimen collection.
